# Immune Tolerance Induction Using Cell-Based Strategies in Liver Transplantation: Clinical Perspectives

**DOI:** 10.3389/fimmu.2020.01723

**Published:** 2020-08-18

**Authors:** Pusen Wang, Zhongyi Jiang, Chunguang Wang, Xueni Liu, Hao Li, Dingyin Xu, Lin Zhong

**Affiliations:** ^1^Department of General Surgery, Shanghai General Hospital, Shanghai Jiao Tong University School of Medicine, Shanghai, China; ^2^Department of Hepatobiliary Surgery, Ruian People's Hospital, Ruian, China

**Keywords:** immune tolerance, liver transplantation, hematopoietic stem cells, regulatory cells, mesenchymal stromal cells

## Abstract

Liver transplantation (LT) has become the best chance and a routine practice for patients with end-stage liver disease and small hepatocellular carcinoma. However, life-long immunosuppressive regimens could lead to many post-LT complications, including cancer recurrence, infections, dysmetabolic syndrome, and renal injury. Impeccable management of immunosuppressive regimens is indispensable to ensure the best long-term prognosis for LT recipients. This is challenging for these patients, who probably have a post-LT graft survival of more than 10 or even 20 years. Approximately 20% of patients after LT could develop spontaneous operational tolerance. They could maintain normal graft function and histology without any immunosuppressive regimens. Operational tolerance after transplantation has been an attractive and ultimate goal in transplant immunology. The liver, as an immunoregulatory organ, generates an immune hyporesponsive microenvironment under physiological conditions. In this regard, LT recipients may be ideal candidates for studies focusing on operative tolerance. Cell-based strategies are one of the most promising methods for immune tolerance induction, including chimerism induced by hematopoietic stem cells and adoptive transfer of regulatory T cells, regulatory dendritic cells, regulatory macrophages, regulatory B cells, and mesenchymal stromal cells. The safety and the efficacy of many cell products have been evaluated by prospective clinical trials. In this review, we will summarize the latest perspectives on the clinical application of cell-based strategies in LT and will address a number of concerns and future directions regarding these cell products.

## Introduction

Liver transplantation (LT) has become the best chance for patients with end-stage liver disease and small hepatocellular carcinoma with chronic liver disease since it was first performed in 1963 (1, 2). With the development of new immunosuppressive regimens and the improvement of surgical techniques, LT has become a routine practice and is increasingly conducted around the world. However, most recipients need open-ended and even lifelong immunosuppression to achieve ideal long-term outcomes. This open-ended immunosuppressive therapy can result in many post-LT complications, such as cancer recurrence, dysmetabolic syndrome, infections, and renal injury. Interestingly, some LT recipients who are taken off immunosuppression for different reasons accidently develop immune tolerance. Among highly selected LT recipients, some of them could discontinue all immunosuppression for more than 1 year while maintaining a stable allograft status, which is defined as “operational tolerance” (3, 4). For these reasons, tolerance of LT has been an attractive and ultimate goal in transplant immunology. Approximately 20% of recipients could become completely tolerant without any immunosuppressant drugs after LT (5–7), whereas such “operational tolerance” is reported only anecdotally in recipients of other organs (8).

The liver has been generally recognized as an immunoregulatory organ (9, 10). It consists in parenchymal and innate immune cells, including hepatocytes and cholangiocytes, liver sinusoidal endothelial cells, hepatic stellate cells, Kupffer cells, stromal cells, liver-derived dendritic cells, natural killer (NK) cells, natural killer T (NKT) cells, and so on (11). The complex interactions between these cells and immune cells contribute to the induction of immune tolerance in the liver (12). The liver receives blood from both the portal vein and the hepatic artery. In the portal vein, the liver confronts various antigens from digested food and the gut microbiome under physiological conditions (13). The immune reaction is tightly controlled and regulated, generating liver-protective immunity while these antigens pass through sinusoids. The responsible mechanisms are associated with various elements, including immature and non-professional antigen-presenting cells, exhausted lymphocytes, transforming growth factor β (TGFβ), and interleukin (IL)-10 in the cytokine milieu, and high proportions of regulatory cells (14). Compared to that in other solid organ transplantations, the incidence of chronic rejection in LT is lower (15). With this background, LT recipients may be ideal candidates for clinical trials studying operative tolerance. A reproducible strategy to induce stable transplant tolerance may achieve success first in LT.

Substantial progress has been made toward immune tolerance induction and in the study of relevant mechanisms in animal models. However, the translation of these strategies into clinical transplantation remains challenging. In addition to novel immunomodulatory drugs such as belatacept, the most promising strategy is cell-based therapy. A variety of cell products were tested to induce tolerance in preclinical experiments, but only a small part of them were evaluated in clinical studies, including induction of chimerism by hematopoietic stem cells and adoptive transfer of regulatory cells and mesenchymal stromal cells. There are many advantages of cell-based strategies, such as low toxicity and long-term efficacy. In addition, a cell-based strategy is expected to control many kinds of inflammatory cells and generate donor antigen-specific tolerance (16). In this review, we mainly focus on cell-based strategies of tolerance induction in LT by clarifying the translational potential of these strategies.

## Hematopoietic Stem Cells for Tolerance Induction

Since the first successful application of mixed chimerism in tolerance induction in human kidney transplantation in 2008 (17), mixed chimerism induced by donor hematopoietic stem cell (HSC) infusion remains one of the most effective treatments for tolerance induction. Chimerism can be defined as tissues from two genetically distinct organisms coexisting in one organism (18). Mixed chimerism, in which both donor and recipient HSCs coexist, leads to donor-specific transplantation tolerance and retains immunocompetence for primary immune responses (19–22). Additionally, mixed chimerism can be induced through a non-myeloablative conditioning protocol, which represents a lower risk and severity of graft-vs.-host disease (GVHD) than the fully allogeneic chimerism induced by myeloablative conditioning.

**Figure 1:**
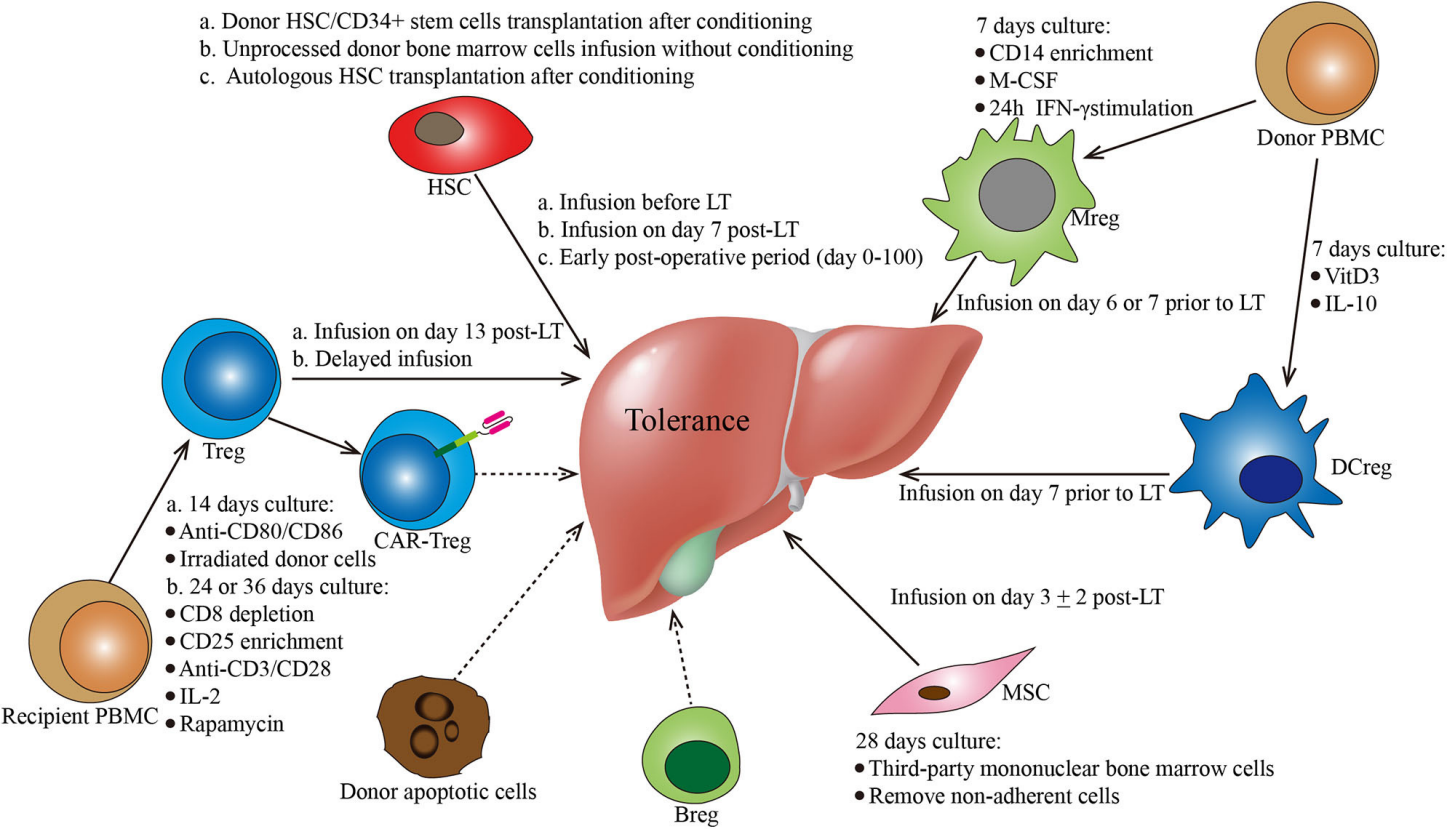
Protocols of various cell-based strategies for tolerance induction in liver transplantation. This figure summarizes the different cell-based therapies described in the manuscript. Cell types, culture conditions, dosages (i.e., cell number), and administration conditions are mentioned for each cell-based therapy. Breg, regulatory B cells; CAR, chimeric antigen receptor; DCreg, regulatory dendritic cells; HSC, hematopoietic stem cells; MSC, mesenchymal stroma cells; PBMC, peripheral blood mononuclear cells; Treg, regulatory CD4+ T cells.

In LT, the application of hematopoietic chimerism to achieve graft tolerance has been studied by various groups. The St. Mary's Hospital group reported that full donor chimerism induced by HSC transplantation could maintain stable allograft tolerance without immunosuppressants. Two patients were immunosuppressant-free with normal liver function for 6 and 7 years (Table 1) (23). Donckier et al. reported two pilot studies in which donor stem cell infusion under non-myeloablative conditioning was used to induce tolerance in living donor liver transplantation (LDLT). Both patients, who were treated with pretransplant conditioning using cyclophosphamide and anti-thymocyte globulin (ATG), discontinued immunosuppressive therapies 90 and 28 days after transplantation without subsequent rejection episodes (Table 1) (24). In the other study, three prospectively enrolled patients were treated with post-transplant conditioning using high doses of ATG and donor CD34^+^ stem cell infusion (5.3–10 × 10^6^ cells/kg) (Figure 1; Table 1). Two of the three recipients successfully discontinued immunosuppression early without subsequent graft deterioration. Of note is that both patients developed acute rejection during follow-up (25). These results are promising. However, among the four immunosuppression-free patients, Donckier et al. reported relatively short follow-ups from 270 days to 561 days. In another study conducted by the University of Miami group, unprocessed donor bone marrow cell infusion without conditioning therapy was investigated for tolerance induction (Figure 1; Table 1). A total of 104 patients of at least 3 years post-transplantation were enrolled, among which 45 patients received donor bone marrow cell infusions (5.94 ± 0.4 × 10^8^ cells/kg) during the early post-operative period and 59 patients did not. Immunosuppressive therapies were tapered slowly over 3 years after their enrollment. Twenty patients, 10 from each group, were immunosuppression-free during follow-up without a significant difference (26). These data indicate that chimerism-based strategies can produce long-term tolerance, but conditioning therapy seems indispensable. In the application of mixed chimerism for tolerance induction in LT, any risk of acute or chronic GVHD should be avoided. Although many studies reported less frequent incidences of GVHD using non-myeloablative conditioning regimen (27), there is still a risk of GVHD with durable mixed chimerism (28, 29). Therefore, modifications of the conditioning protocol and maybe delayed HSC infusion might be important next steps for chimerism-induced tolerance in LT.

**Table 1:** Clinical studies/trials of cell-based strategies for tolerance induction in LT.

**Cell type**	**Sample size/stage**	**Cell dose (total)**	**Reference/trial ID**
**HSC**			
Allogeneic, HSC transplantation	2 cases		23
Purified donor CD34^+^ stem cells	2 cases	3.3–5.7 × 10^6^ cells/kg	24
Purified donor CD34+ stem cells	3 cases	5.3–10 × 10^6^ cells/kg	25
Donor bone marrow cells	104 cases	5.94 ± 0.4 × 10^8^ cells/kg	26
Autologous, HSC transplantation	Phase II		NCT02549586
**Treg**			
Autologous, donor alloantigen-specific specific Treg	Phase I	0.34–6.37 × 10^6^ cells/kg	46, UMIN-000015789
Autologous, polyclonal Treg	Phase I/II	0.5–4.5 × 10^6^ cells/kg	47, NCT02166177
Autologous, donor alloantigen-specific specific Treg	Phase I	1 × 10^6^ cells/kg	NCT01624077
Autologous, donor alloantigen-specific specific Treg	Phase I/II	1–2.5 × 10^6^ cells/kg	NCT03577431
Autologous, donor alloantigen-specific specific Treg	Phase I	25–960 × 10^6^ cells/kg	NCT02188719
Autologous, donor alloantigen-specific specific Treg	Phase I/II	300–500 × 10^6^ cells/kg	NCT02474199
**DCreg**			
Donor DCreg (infusion at 7 days before LT)	Phase I/II	2.5–10 × 10^6^ cells/kg	NCT03164265
Donor DCreg (infusion at 7 days prior to IS weaning)	Phase I/II		NCT04208919
**MSC**			
Third-party BM-derived MSC	Phase I	1.9–2.7 × 10^6^ cells/kg	91, NCT01429038
Third-party BM-derived MSC	Phase I	1–2 × 10^6^ cells/kg	NCT02260375
Allogeneic, MSC	Phase I/II	6 × 10^6^ cells/kg	NCT02706132
Umbilical cord derived MSC	Phase I	3 × 10^6^ cells/kg	NCT01690247
Donor BM-derived MSC (pediatric LT)	Phase I	2 × 10^6^ cells/kg	NCT02957552

*LT, liver transplantation; HSC, hematopoietic stem cell; Treg, regulatory T cell; DCreg, regulatory DC cell; IS, immunosuppression; MSC, mesenchymal stromal cell; BM, bone marrow*.

In addition to donor HSC infusion, autologous hematopoietic stem cell transplantation (HSCT) has also been studied for tolerance induction. Autologous HSCT may reset the deregulated immune system into a tolerant status by regenerating new autotolerant T and B cells and increasing immune regulatory mechanisms (30–32). The therapeutic efficacy of autologous HSCT for various autoimmune diseases has been reported by clinical trials, such as trials in systemic and multiple sclerosis (33–35), Crohn's disease (36), and scleroderma (37). In LT, there is an ongoing clinical trial evaluating the ability of autologous HSCT to induce tolerance (NCT02549586) (Figure 1; Table 1). The researchers plan to recruit 10 liver transplant recipients. The purified HSCs will be infused following a chemotherapy- and ATG-based conditioning regimen. The immunosuppressive drugs will be withdrawn at 6 months post-HSCT.

## Regulatory T Cells for Tolerance Induction

Regulatory T cells (Tregs), commonly distinguished into natural Tregs (nTregs) and inducible Tregs (iTregs) (38), are a population of CD4+ cells that constitutively express the Forkhead box P3 (Foxp3) transcription factor. In 2001, human Tregs were initially identified as CD4+CD25+ T cells (39), which comprise 5–10% of total peripheral CD4+ T cells (40). Over the past two decades, Tregs have been studied and are known to be responsible for maintaining immune homeostasis and tolerance (41, 42). The adoptive transfer of Tregs has been successful and proven effective in murine (43, 44) and non-human primate transplantation models (45).

In the LT setting, tolerance induction by Treg infusion has been reported in two clinical trials to date (46, 47). Researchers from Hokkaido University reported a pilot study of tolerance induction with Treg-based cell therapy in living donor LT in 2016 (46). In this study, donor-antigen-specific iTregs were obtained *ex vivo* by coculturing recipient lymphocytes with irradiated donor cells and anti-CD80/CD86 mAbs for 2 weeks. At day 13 after LT, the expanded cells were administered to the recipients at a mean dose of 3.39 × 10^6^/kg CD4^+^CD25^+^Foxp3^+^ cells (Figure 1; Table 1). This dose is much lower than the dose of nTregs for transplant cell therapy since donor-antigen-specific iTregs are considered to be more potent than nTregs (48, 49). The infusion caused no significant adverse events. After infusion, the immunosuppressive agent weaning program was initiated at 6 months post-LT and completely discontinued at 18 months. Among the 10 consecutively enrolled patients, seven completely stopped their immunosuppressive regimen for 16–33 months with normal graft function and histology. The other three recipients who had autoimmune liver diseases developed acute cellular rejection and resumed reduced doses of immunotherapy. This is the first study of successful operational tolerance induction using the adoptive transfer of Tregs in LT.

More recently, the King's College London group published the results of their phase I clinical trial, ThRIL, evaluating the safety and the efficacy profile of Treg therapy in LT recipients (47). In this trial, patients with an autoimmune disease were excluded. Tregs isolated from the recipients were expanded under polyclonal conditions *ex vivo* using anti-CD3/CD28 beads, IL-2, and rapamycin for 24 or 36 days. Three patients awaiting LT were enrolled and received an infusion of 1 × 10^6^ Tregs/kg 83–110 days post-transplant, while the other six patients were recruited 6–12 months post-transplant and received an infusion of 4.5 × 10^6^ Tregs/kg 112–151 days after enrollment (Figure 1; Table 1). Of note is that one of the patients who received an infusion of 4.5 × 10^6^ Tregs developed a fever of >39°C associated with rigors, which was classified as a dose-limiting toxicity. In general, this autologous non-specific Treg transfer was considered to be safe and exerted potentially beneficial donor-specific immunosuppressive effects.

Treg-induced immune regulation is the best-studied and core mechanism of tolerance. Both preliminary clinical studies demonstrated the safety and the efficacy of Treg strategies in human LT, which has great potential for future clinical translation. Many other registered phase I/II clinical trials assessing the safety and the efficacy of Treg infusion are in progress (NCT01624077, NCT03577431, NCT02188719, and NCT02474199) (Table 1). However, multicenter studies with large sample sizes need to be conducted, and future studies should focus on the protocol of Treg infusion, such as cell dosage, timing/frequency of infusion, optimal immunosuppressive regimen, and its late complications. Additionally, some other approaches to generate antigen-specific Tregs can be promising in LT, such as chimeric antigen receptor (CAR) transduction (50). It was shown that the adoptive transfer of Tregs engineered with a CAR which targets HLA-A2 can suppress skin allograft rejection in humanized mouse models (51, 52). Therefore, these modified cells may have a great potential in LT.

## Regulatory Dendritic Cells for Tolerance Induction

Dendritic cells (DCs) were first identified by Steinman and Cohn in 1973 (53, 54) and have proved potent antigen-presenting cells linking the innate and the adaptive immune responses (55). Over the following years, based on their morphological features, ontogenies, locations, and functions, various DC subsets have been identified, including conventional DCs (cDCs), plasmacytoid DCs (pDCs), Langerhans cells (LCs), and inflammatory DCs (56, 57). It has been reported that DCs can be either immunogenic or tolerogenic in different states (58, 59). Of note is that the ablation of DCs could break the self-tolerance of CD4^+^ T cells and result in spontaneous fatal autoimmunity (60).

Tolerogenic DCs or regulatory DCs (DCregs) are characterized by a low expression of MHC gene products (MHC class I and II) and co-stimulatory molecules (CD80 and CD86) and a high expression of co-inhibitory ligands (PD-L1) and death-inducing ligands (FasL). In terms of functions, DCregs are resistant to maturation, able to produce anti-inflammatory cytokines (IL-10 and TGFβ), and impair T cell proliferation.

In LT, tolerance induction by DCregs is ongoing as phase I/II clinical trials conducted by the University of Pittsburgh group (61). Actually, the safety, the tolerability, and the feasibility of autologous DCreg infusion have been confirmed by various clinical studies in autoimmune disorders, such as rheumatoid arthritis (62, 63), type 1 diabetes (64), and Crohn's disease (65). In a preclinical non-human primate renal transplant model, it has been demonstrated that a single pretransplant DCreg infusion can prolong MHC-mismatched renal allograft survival (66–68). In an ongoing trial (NCT03164265), according to preclinical experience, DCregs are generated from monocytes of prospective living donors in the presence of VitD3 and IL-10. Then, the expanded cells are infused at a dose of 2.5–10 × 10^6^/kg (54) into the respective recipients 7 days before transplantation (Figure 1; Table 1). Weaning and withdrawal of the immunosuppressive drugs begin at 12 and 18 months post-transplantation, respectively.

More recently, the University of Pittsburgh group registered another clinical trial evaluating the delayed infusion of DCreg for tolerance induction in LDLT (NCT04208919) (Table 1). In this clinical trial, recipients who are between 1 and 3 years after transplantation will be enrolled. Those enrolled patients will receive a single infusion of donor-derived DCreg. At 1 week after that, immunosuppression weaning will be initiated slowly. Since preclinical experience to date has proven that DCreg infusion is safe, the results of their clinical trials are awaited with great interest.

## Regulatory Macrophages for Tolerance Induction

Macrophages are immune cells of hematopoietic origin that provide crucial innate immune defense and have tissue-specific functions in the regulation and the maintenance of organ homeostasis (69). Various macrophage subsets with distinct functions have been identified, including classically activated macrophages (M1 macrophages), alternatively activated macrophages (M2 macrophages), regulatory macrophages (Mregs), tumor-associated macrophages, and myeloid-derived suppressor cells (70–73).

Human Mregs reflect a unique state of macrophage differentiation, distinguished from macrophages in other activation states by their particular mode of derivation, robust phenotype, and potent T cell-suppressing function (74). According to the protocol from University Hospital Regensburg (74, 75), Mregs could be generated from CD14^+^ blood monocytes in the presence of M-CSF and a further 24-h stimulation with IFN-γ after 7 days of culture. In terms of cell surface phenotype, these cells are homogeneously CD14^−/low^HLA-DR^+^CD80^−/low^CD86^+^CD16^−^TLR2^−^CD163^−/low^ (75). In this pilot study, these cells were administered 1 week prior to transplantation to two living-donor renal transplant recipients at doses of 7.1 × 10^6^ and 8 × 10^6^ cells/kg (75) (Figure 1). Both patients were weaned to low-dose tacrolimus monotherapy and maintained normal graft function. Of note is that the infused cells were ^111^In-labeled, which is different from that of other studies. The tracking results showed that the donor-derived Mregs remained viable for more than 30 days and migrated from the pulmonary vasculature *via* the blood to the liver, spleen, and bone marrow. The clinical trial of Mreg treatment for renal transplantation was registered as the One study, in which donor-derived Mregs (2.5–7.5 × 10^6^ cells/kg) were infused 6–7 days before transplantation into recipients of a living donor renal transplant (NCT02085629). However, in LT, there is no registered clinical trial about Mregs to date.

## Mesenchymal Stromal Cells for Tolerance Induction

Mesenchymal stromal cells (MSCs) are plastic-adherent non-hematopoietic multipotent cells that are able to differentiate into osteoblasts, adipocytes, and chondroblasts under standard *in vitro* differentiating conditions (76). In terms of cell surface phenotype as measured by flow cytometry, these cells express CD105, CD73, and CD90 and lack expression (<2% positive) of CD45, CD34, CD14, or CD11b, CD79a or CD19, and HLA class II (76, 77). Bone marrow was first and most commonly used to isolate MSCs for clinical applications since 2004 (78). Over the last few years, other sources of MSCs have been found and proposed for clinical use, including adipose tissue (79), dental tissues (80), placenta (81), umbilical cord tissue (82), and cord blood (83). MSC-induced immunosuppression targets both the innate (84) and the adaptive immune systems (85). A variety of studies documented the potent immunosuppressive capacity of MSCs *in vitro* and *in vivo* (86–88). The immunoregulatory effect of MSCs is dose dependent and seems independent of MHC (89). The effects of autologous MSC infusion have been evaluated in kidney transplantation in a randomized controlled trial with a large sample size (90). In this study, MSC infusion resulted in a lower incidence of acute rejection, a lower risk of opportunistic infection, and better renal function at 1 year.

In LT, MSC therapy has been evaluated in a phase I–II clinical study (91). In this study, MSCs were generated by isolating mononuclear BM cells with Ficoll and expanding them in a 4 week culture. Third-party unrelated MSCs were infused at a dose of 1.5–3 × 10^6^/kg on post-transplantation day 3 ± 2 in 10 patients (Figure 1; Table 1). Compared with 10 control liver transplant recipients, patients who received MSC infusion did not have an impairment of organ functions or an increased rate of opportunistic infection or malignancies. Weaning and withdrawal of the immunosuppressive drugs were attempted from month 6 to 12 post-transplantation. Among nine MSC recipients, tacrolimus and mycophenolate mofetil withdrawal were successfully achieved in only one patient, while the other eight patients failed due to either graft rejection or a significant increase in transaminases.

The very fast tapering of immunosuppressive drugs within 3 months might explain the failure of tolerance induction using MSCs in this study (91, 92). MSC-induced immunoregulation might be disrupted by very fast drug discontinuation, which could promote effector T cell activation (92). In addition, lack of induction therapy, insufficient MSC dosage, timing of infusion, infusion routes, and different sources might also account for the failure. As far as we know, MSC infusion for tolerance induction in LT is being studied by various other registered clinical trials (NCT02260375, NCT02706132, NCT01690247, and NCT02957552) (Table 1), including that using donor-derived MSC and that using umbilical-cord-derived MSC. Indeed these variables should be studied in future studies to achieve better results.

## Regulatory B Cells for Tolerance Induction

Regulatory B cells (Bregs) are immunosuppressive cells that support immunological tolerance (93, 94). Bregs express immune-regulatory cytokines, including IL-10, TGF-β, and IL-35, through which Bregs can suppress the differentiation of various pro-inflammatory lymphocytes (93, 94).

The role of Bregs in transplant tolerance has been studied mostly in rodents using heart (95, 96) and islet (97, 98) graft models. Stable immune tolerance to solid organ and islet cell grafts can be induced using antibodies directed at CD45RB, and this tolerance is dependent on Bregs (95, 97, 98). Furthermore, graft rejection in B cell-deficient mice after islet transplantation could be reversed to tolerance by the adoptive transfer of B cells from tolerant mice (97). The mechanisms of Breg-induced tolerance remain unclear, and research is still active. Breg-dependent islet transplant tolerance also requires the functions of natural killer cells and Tregs (98). Although promising, many questions remain unsolved in Breg-based strategies, such as the method of *ex vivo* culture and the expansion and the ability of tolerance induction in large animals. The field of Breg-induced tolerance is very immature and has a long way to go before translation into clinical LT.

## Apoptotic Cells for Tolerance Induction

Apoptosis is a genetically programmed cell death mechanism occurring during the elimination of unwanted or dangerous cells. Apoptotic cells exhibit immunomodulatory properties through various mechanisms, including inhibiting pathogenic T or B cell responses and inducing pro-tolerogenic/regulatory cells (99). In addition to those specific cell populations, apoptotic cell-based therapy is another promising strategy for tolerance induction in transplantation. In many animal studies, apoptotic cells, mainly apoptotic splenocytes in transplantation setting, favor the engraftment of liver (100, 101), cardiac (102, 103), islet (104), and hematopoietic (105) allografts.

In LT, tolerance induction using apoptotic cells was studied in rats by two groups. Researchers from Zhejiang University reported that donor apoptotic cells can promote liver graft acceptance using a rat LT model (106). In this study, apoptotic splenic lymphocytes induced by ultraviolet-C (UVC) irradiation at a dosage of 5 × 10^7^ cells/rat were infused intravenously at 7 days before LT. In terms of mechanism, they found increased peripheral blood Tregs in rats treated with UVC-irradiated lymphocytes. In another study performed by the Zhejiang University group, they showed that the combination of tolerogenic DCs and apoptotic lymphocytes alleviates rejection after LT (100). The other group, Zhejiang Cancer Hospital group, also reported that preinfusion of apoptotic lymphocytes can induce immune tolerance in a rat LT model (101). The apoptotic cells used in this study were obtained from donor peripheral blood. Lymphocytes irradiated by X-ray from an electron linear accelerator at an absorbed dose of 2.0 Gy were infused intravenously at a dosage of 5 × 10^7^ cells/rat 7 days before operation. However, there are no clinical studies or studies using nonhuman primate transplantation models considering the immunoregulatory role of apoptotic cells in LT. This can be an important next step in the field.

## Concerns

There are many concerns for cell-based therapies. First, for allogenic/autologous HSC infusion, intense myeloablative or non-myeloablative conditioning therapy may not be tolerated by patients with end-stage liver disease. Of note is that, in the absence of conditioning therapy, donor HSC infusion may show no significant effect in LT (26). Various non-myeloablative conditioning regimens of reduced intensity are now being studied, revised, and improved to induce tolerance with less toxicity (27, 107–109). Second, protocols of these regulatory cell-based therapies are not clearly established. The optimal dose of regulatory cells could be highly important for those strategies using non-donor-specific therapies. Overdose may result in original disease recurrence, infection, and other immunosuppression-related complications. In addition, there is no current consensus regarding the method and the timing of administration. The potential influence of different protocols needs to be observed in a study with long follow-up. Third, the purity of *in vitro*-expanded cells represents another significant concern. After stimulation, culture, and expansion, cell products without classical confirmed markers cannot be purified. There are subpopulations in these cell products that have different functions, such as resting and activated Tregs, highly suppressive Tregs, and non-Treg FoxP3^+^ cells (110–113). Additionally, Tregs can acquire the expression of transcription factors associated with effector T cell programs (called Th-like Tregs), which is called Treg cell plasticity (114). These Th-like Tregs can be pro-inflammatory rather than suppressive, which play an opposite role in tolerance induction. Fourth, the stability of infused cells has not been determined. Except for one study (75), none of the studies thus far have used cell products that were radioactively labeled. Without genetic or radioactive labels, *in vivo* homing and the stability of infused cells are difficult to detect. Fifth, at least for now, another drawback of cell-based therapy is the cost. Basic researches, clinical trials, and products of these cell-based therapies are very costly. Last but not least, the selection of patients is one of the top issues. Allogenic/autologous HSC infusion may not be conducted in patients with end-stage organ disease considering the toxicity of recipient conditioning (115, 116). Regulatory cells may play a detrimental role in patients with hepatitis B infection (117, 118), hepatitis C infection (119, 120), or hepatocellular carcinoma (121, 122). Pilot studies or phase I/II clinical trials with small sample sizes are unable to uncover all potential risks. It is vital to conduct high-quality studies with large sample sizes to assess the safety and the efficacy of these cellular strategies in tolerance induction.

## Summary and Outlook

Undoubtedly, cell-based strategies have a great potential in tolerance induction in transplantation. In particular, LT provides a great opportunity to achieve this goal because of an immunoregulatory microenvironment in the liver. Compared to immunosuppressant drugs, cell products can generate donor-specific tolerance with low toxicity and long-term efficacy. Theoretically, they could maintain normal graft function without immunosuppression and keep the protective properties of the immune system intact. Various cell products using different infusion protocols are being evaluated in clinical studies (Figure 1). These preliminary clinical studies have demonstrated a promising breakthrough in tolerance induction using cell-based therapies, including HSCs, Tregs, Mregs, DCregs, and MSCs. Breg-induced tolerance has mostly been studied in animal models, and the field is still in its infancy. The clinical studies or trials exploring the safety and the efficacy of cell-based strategies for tolerance induction in LT are listed in Table 1. Many excellent translational results may show up in the next few years. However, various problems with these strategies remain challenging. These strategies are not equally effective in each patient, suggesting that diverse mechanisms of immune tolerance exist among different individuals. Identifying useful biomarkers of immune tolerance that could guide the gradual tapering of immunosuppression after cell-based therapies is necessary. In addition, the heterogeneity of immunological status and health state between patients makes tolerance induction difficult with a universal protocol. The combination of cell products and other therapies as well as individualized treatment might provide ideal results. However, more high-quality clinical studies focusing on these strategies need to be performed for practical translation from bench to bedside.

## Author Contributions

PW and ZJ wrote this manuscript and prepared the table and the figure. LZ and DX devised and supervised this project. CW, XL, and HL assisted in the literature search and manuscript editing. All authors contributed to the article and approved the submitted version.

## Conflict of Interest

The authors declare that the research was conducted in the absence of any commercial or financial relationships that could be construed as a potential conflict of interest.
